# Trends in ART Initiation among Men and Non-Pregnant/Non-Breastfeeding Women before and after Option B+ in Southern Malawi

**DOI:** 10.1371/journal.pone.0165025

**Published:** 2016-12-21

**Authors:** Kathryn Dovel, Sara Yeatman, Joep J. van Oosterhout, Adrienne Chan, Alfred Mantengeni, Megan Landes, Richard Bedell, Gift Kawalazira, Sumeet Sodhi

**Affiliations:** 1 Center for World Health, Division of Infectious Diseases, Department of Medicine, David Geffen School of Medicine, University of California Los Angeles, Los Angeles, United States of America; 2 Department of Health and Behavioral Sciences, University of Colorado Denver, Denver, United States of America; 3 Department of Medicine, University of Malawi College of Medicine, Blantyre, Malawi; 4 Dignitas International, Zomba, Malawi; 5 Department of Family and Community Medicine, University of Toronto, Toronto, Canada; 6 Division of Infectious Diseases, Department of Medicine, Sunnybrook Health Sciences Centre, University of Toronto, Toronto, Canada; 7 District Health Office, Ministry of Health, Zomba, Malawi; 8 Department of Family and Community Medicine, University Health Network, Toronto Western Hospital, Toronto, Canada; Katholieke Universiteit Leuven Rega Institute for Medical Research, BELGIUM

## Abstract

**Background:**

Option B+ is promoted as a key component to eliminating vertical transmission of HIV; however, little is known about the policy’s impact on non-targeted populations, such as men and non-pregnant/non-breastfeeding women. We compare ART uptake among non-targeted populations during pre/post Option B+ periods in Zomba District, Malawi.

**Methods:**

Individual-level ART registry data from 27 health facilities were digitized and *new* ART initiates were disaggregated by sex and type of initiate (Option B+ or not). Data were analyzed over the pre- (January 2009-June 2011) and post- (July 2011- December 2013) Option B+ periods.

**Results:**

After the implementation of Option B+, the total number of new female initiates increased significantly (quarterly median: 547 vs. 816; *P* = 0.001) and their median age decreased from 34 to 31 years (*P* = <0.001). Both changes were the result of the rapid and sustained uptake of ART among Option B+ clients. Post-policy, Option B+ clients represented 48% of all new female initiates while the number of females who initiated through CD4 or WHO staging criteria significantly decreased (quarterly median: 547 vs. 419; *P* = 0.005). The number and age of male initiates remained stable; however, the proportion of men among new initiates decreased (36% vs. 31%; *P* = <0.001).

**Conclusions:**

Option B+ shifted the profile of first-time initiates towards younger and fertile women. Declines among non-Option B+ women most likely reflect earlier initiation during pregnancies before deteriorations in health. The decreased proportion of men among first-time initiates represents a growing gender disparity in HIV services that deserves immediate attention.

## Introduction

In 2011, Malawi became the first country to implement Option B+, a policy that provides immediate and lifelong antiretroviral therapy (ART) to all pregnant and breastfeeding women who test HIV positive [1]. The policy has been heralded as a major success [2] and has been rapidly adopted throughout sub-Saharan Africa (SSA). As of 2015, 20 countries in SSA had implemented Option B+ policies [3].

Despite its widespread implementation, it remains unclear how Option B+ affects the landscape of ART for non-targeted populations such as men and non-pregnant/non-breastfeeding women. Some scholars have questioned the ethics of Option B+ since it prioritizes test-and-treat strategies for pregnant women while neglecting universal access for other populations [4–6]. This may be particularly detrimental for men who are already underrepresented in HIV services [7,8] and thus, at increased risk of AIDS-related mortality [9]. Despite high ART coverage among pregnant/breastfeeding women through Option B+, HIV will not be eliminated unless high coverage is also reached for the broader population [10–12]. Therefore, the question of if and how Option B+ affects ART uptake among non-targeted populations is of critical public health importance [4,9]. We use individual-level ART registry data from Zomba District, Malawi to compare trends in ART uptake before and after the implementation of Option B+, paying particular attention to ART initiation among non-targeted populations.

## Methods

### Setting

In July 2011, Malawi began the rapid rollout of Option B+. Under Option B+, over 50% of facilities in southeastern Malawi allowed newly diagnosed pregnant women to initiate ART at antenatal care clinics, improving the linkage between testing and treatment [13]. At the same time, eligibility criteria for ART expanded from CD4 count <250 cells /mm^3^ to CD4 count ≤350 cells/mm^3^ in addition to the standard WHO Stage 3/4 criteria [14,15]. Access to CD4 machines remained limited, however, with functioning CD4 machines only available at 11% of ART sites in the country as of mid-2011 and 22% by 2014 [16,17].

Zomba district is located in southeastern Malawi, where Dignitas International, a Canadian medical and research organization has a long-term presence. ART services are primarily provided by Ministry of Health facilities and faith-based facilities that offer services at minimal cost. Private facilities are scarce and see only a small number of clients.

### Data Collection

Data come from routine ART registers that were digitized as part of the Zomba Observational Cohort Study [18]. The cohort contains individual-level data including sex, age, date of ART initiation, reason for initiation (Option B+, WHO staging, or CD4 count), and type of ART initiate (clients who start ART for the first-time, re-initiates who previously defaulted from ART, or transfer clients). Methods for collecting data are described in detail elsewhere [19]. Registers were kept in ART counseling rooms and reviewed quarterly by the Ministry of Health and Dignitas International for quality assurance and monitoring purposes [20]. In March 2011, 27 of the district’s 28 non-private ART sites were included in the Zomba Observational Cohort Study. Decentralization of ART in the district had begun by 2008 and by 2011 most health facilities already offered ART [19].

### Statistical Analysis

Clients were included in the analysis if they were first-time initiates, initiated ART between January 1^st^ 2009 and December 31^st^ 2013, and were aged ≥15 years. Because pregnancy status prior to Option B+ was not systematically recorded, we categorize female clients as Option B+ or non-Option B+ initiates. Women were considered Option B+ initiates if they qualified for ART solely because they were pregnant or breastfeeding. Women who met CD4 or WHO staging criteria were considered non-Option B+ initiates, even if pregnant or breastfeeding. CD4 count at time of ART initiation was excluded due to missing data across the study period.

Sex was missing for 0.01% of all cases and discrepancies in reason for initiation existed for 0.4% of all cases. These cases were dropped from analyses (n = 123). Data were collapsed by sex and indication for ART initiation and were aggregated into quarters. We use Wilcoxon rank-sum tests to determine differences between the pre- and post-Option B+ periods. Ethical approval was received from the National Health Services Research Committee, Malawi and the University Health Network, Toronto, Canada.

## Results

**Fig 1:**
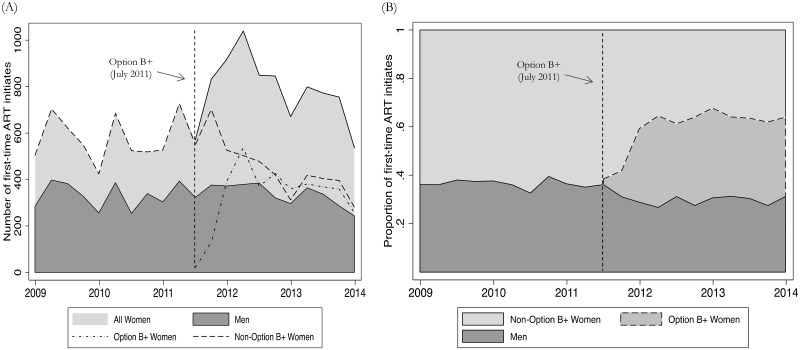
Quarterly trends in new ART initiates by gender and indication for ART: absolute numbers (Panel A) and proportions (Panel B).

In total, 24,164 clients were included over the 5-year study period. The median number of female initiates per quarter increased by 39% in the 2.5 years after the implementation of Option B+ (July 2011-December 2013) compared to the 2.5 years (January 2009-June 2011) preceding the policy (quarterly median: 547 vs. 816; *P* = 0.001; Table 1). The increase was due to the rapid and sustained uptake of treatment among Option B+ clients. In the years following the policy, Option B+ clients comprised 48% of all female initiates (Fig 1, Panel B). The median number of women initiating ART through CD4 or WHO criteria (non-Option B+ women) per quarter decreased by 23% following policy implementation, despite the CD4 threshold change from 250 to 350 cells/mm^3^ (quarterly median: 547 vs. 419; *P* = 0.005). The greatest decline for non-Option B+ women was seen in the latter half of 2012, approximately one year after the initial rollout of Option B+ (Fig 1, Panel A). In contrast, the median number of first-time male initiates did not change significantly after the introduction of Option B+ (quarterly median: 327 vs. 351; *P* = 0.833). Nonetheless, men’s representation among new initiates declined over the study period from 36% the quarter before implementation to a low of 27% by the first quarter of 2012. Overall, there was a 14% decrease in the proportion of first-time initiates who were men following policy implementation (median quarterly percent of first-time initiates who were male: 36% vs. 31%; *P*<0.001).

**Table 1:** Changes in first-time initiates by gender and time of ART initiation: pre- and post-Option B+.

Variable	Pre-Option B+ January Jan 2009–March 2011	Post-Option B+ January July 2011–Dec 2014	*P*
Median number of first-time initiates per quarter	874	1165	0.007
Men	327	351	0.833
All women	547	816	0.001
Option B+ women	-	370	-
Non-Option B+ women	547	419	0.005
Median percentage of first-time initiates per quarter			
Men	36	31	<0.001
All women	64	69	<0.001
Option B+ women	-	48	-
Non-Option B+ women	100	52	<0.001
Median age of new initiates (IQR)			
Men	37 (32–45)	38 (32–45)	0.596
All women	34 (28–41)	31 (26–38)	<0.001
Option B+ women	-	29 (24–33)	-
Non-Option B+ women	34 (28–41)	35 (29–43)	0.003

The median age of female initiates significantly decreased post-Option B+, from 34 years (IQR: 28–41 years) to 31 years (IQR: 26–38 years; *P<*0.001; Table 1). This decrease was due to the younger age of Option B+ initiates (median age for Option B+ initiates was 29 years; IQR: 24–33 years). The age of female non-Option B+ initiates increased over the study period [34 years pre-Option B+ (IQR: 28–41 years) vs. 35 years post-Option B+ (IQR: 29–43 years); *P* = 0.003]. Men’s median age at initiation remained stable throughout the study period [38 years pre-Option B+ (IQR: 32–45 years) vs. 38 years post-Option B+ (IQR: 32–45 years); *P* = 0.596].

## Discussion

In Zomba District, as with the whole of Malawi [2], the total number of first-time ART initiates increased considerably following the implementation of Option B+. Despite the corresponding widening of CD4 eligibility criteria, this increase was due exclusively to pregnant/breastfeeding women initiating though Option B+. The number of men initiating ART for the first time remained stable over the study period, while the number of women initiating due to advanced HIV infection declined.

There are two explanations for why non-targeted populations did not see an increase in first-time initiates. First, access to CD4 machines remained low [17], which limited the impact of the lowered CD4 threshold for ART eligibility because, in practice, the new CD4 criteria could not be implemented in most settings [21]. Second, widening CD4 criteria was not coupled with improved diagnostic or linkage systems. Even if more people were eligible, they may not have started ART. For example, a recent study from South Africa showed that without additional support, clients with higher CD4 counts are less likely to link to care than those with lower CD4 counts [22]. Other studies have found that despite widened eligibility criteria, the mean CD4 count at initiation across SSA has not increased [23].

The decline in first-time initiations among women who were eligible through CD4 or WHO staging criteria may signal the success of Option B+. Given the frequency with which women are pregnant in Malawi (where the total fertility rate is 5.7 children [24]) and high attendance for antenatal care (approximately 95% of pregnant women attend at least one antenatal visit [17,24]), women of reproductive age are likely to initiate ART via Option B+ before their health deteriorates. Thus, Option B+ could effectively benefit the majority of women living with HIV since most women become infected between 20–29 years of age, the peak years of fertility [24]. The overlap between age of HIV infection and age of peak fertility may account for the decline in women initiating due to ill health after Option B+, and the concurrent aging of this client population. Notably, women excluded from the benefits of Option B+ include infertile women, those who choose not to get pregnant because of their HIV infection, those who do not use antenatal services, and those who become infected later in life.

Beyond absolute changes in ART initiates, it is important to consider questions of equity in access given ART's role in prolonging the health of infected individuals and reducing HIV transmission [25,26]. Although men’s uptake of treatment remained stable throughout the study period, gender differences increased among first-time initiates. Given that men comprise over 40% of all HIV infections in Malawi [27], their decline from 36% to 31% of first-time initiates reflects a concerning underrepresentation of men on ART.

There are several limitations that should be noted. First, our study relies on operational, routine ART monitoring tools. Given high workloads, some errors and missing data are unavoidable. Regular supervision by experienced programme staff minimizes inaccuracy [20], and it is unlikely that systematic errors exist throughout multi-site data. Second, implementation of CD4 counting and WHO clinical staging for pregnant women may have become less stringent after the implementation of Option B+, potentially classifying women who would be eligible for ART due to ill health as Option B+ clients. Third, we attribute changes in ART initiations after 2011 to Option B+ and changing eligibility criteria; however, it is possible that other changes at the policy- or community-level contributed to differences observed. Finally, we did not examine ART outcomes. Other studies in Malawi have shown higher loss-to-follow-up among Option B+ women than other initiates [28–30], so there is need to measure and examine the long-term health benefits of Option B+ for women.

Drawing on the Malawi experience, we find that Option B+ is particularly beneficial for fertile women living with HIV, not just those who are currently pregnant/breastfeeding. While the policy did not negatively impact absolute numbers of men initiating treatment, it widened gender disparities. If implemented similarly, other countries adopting Option B+ could expect widening gender disparities in first-time ART enrollees and lower numbers of women initiating treatment due to advance HIV infection. In general, the latter is a good thing as women will be targeted and treated earlier, which will improve their health [31] and reduce transmission to their children and partners [32,33]. If, however, this trend leads to a divestment in CD4 and clinical staging services, then men and sub-fertile women, for whom these remain their only points of entry into treatment, will be disadvantaged.

Fortunately, recent WHO guidelines now recommend universal treatment strategies [34] and many countries are adopting the new guidelines [35]. However, there is reason to be skeptical that universal treatment will ameliorate gender disparities in HIV services. Across the region men are less likely to be tested for HIV [36,37] and present into care at later stages of AIDS [38–40]. Unless men are brought into care earlier, only a small proportion of those tested will benefit from widened eligibility criteria. Countries maintaining Option B+ and those transitioning to universal treatment should consider targeted strategies to better engage men and sub-fertile women. Potential strategies include improving provider-initiated testing for services in addition to antenatal care [41], increasing access to community-based testing through innovations such as self-testing [42], strengthening existing linkage systems, and implementing community-based approaches throughout the continuum of care [43–45]. Additionally, routine data should be collected by gender and pregnancy/breastfeeding status to allow for the monitoring of trends during the rapid shifts in policy.
